# Process Development for Newcastle Disease Virus-Vectored Vaccines in Serum-Free Vero Cell Suspension Cultures

**DOI:** 10.3390/vaccines9111335

**Published:** 2021-11-16

**Authors:** Julia Puppin Chaves Fulber, Omar Farnós, Sascha Kiesslich, Zeyu Yang, Shantoshini Dash, Leonardo Susta, Sarah K. Wootton, Amine A. Kamen

**Affiliations:** 1 Viral Vectors and Vaccines Bioprocessing Group, Department of Bioengineering, McGill University, Montreal, QC H3A 0G4, Canada; julia.puppinchavesfulber@mail.mcgill.ca (J.P.C.F.); omar.farnosvillar@mcgill.ca (O.F.); sascha.kiesslich@mail.mcgill.ca (S.K.); zeyu.yang2@mcgill.ca (Z.Y.); shantoshini.dash@mail.mcgill.ca (S.D.); 2 Department of Pathobiology, Ontario Veterinary College, University of Guelph, Guelph, ON N1G 2W1, Canada; lsusta@uoguelph.ca (L.S.); kwootton@uoguelph.ca (S.K.W.)

**Keywords:** Newcastle Disease Virus, Vero suspension culture, viral vaccine bioprocess, bioreactor production, vaccine production platform, COVID-19, SARS-CoV-2

## Abstract

The ongoing COVID-19 pandemic drew global attention to infectious diseases, attracting numerous resources for development of pandemic preparedness plans and vaccine platforms—technologies with robust manufacturing processes that can quickly be pivoted to target emerging diseases. Newcastle Disease Virus (NDV) has been studied as a viral vector for human and veterinary vaccines, but its production relies heavily on embryonated chicken eggs, with very few studies producing NDV in cell culture. Here, NDV is produced in suspension Vero cells, and analytical assays (TCID_50_ and ddPCR) are developed to quantify infectious and total viral titer. NDV-GFP and NDV-FLS (SARS-CoV-2 full-length spike protein) constructs were adapted to replicate in Vero and HEK293 suspension cultures using serum-free media, while fine-tuning parameters such as MOI, temperature, and trypsin concentration. Shake flask productions with Vero cells resulted in infectious titers of 1.07 × 10^8^ TCID_50_/mL for NDV-GFP and 1.33 × 10^8^ TCID_50_/mL for NDV-FLS. Production in 1 L batch bioreactors also resulted in high titers in culture supernatants, reaching 2.37 × 10^8^ TCID_50_/mL for NDV-GFP and 3.16 × 10^7^ TCID_50_/mL for NDV-FLS. This shows effective NDV production in cell culture, building the basis for a scalable vectored-vaccine manufacturing process that can be applied to different targets.

## 1. Introduction

Infectious diseases are present throughout history, emerging and reemerging as decades pass [1]. In the most recent years, the world has seen outbreaks of H1N1 influenza, severe acute respiratory syndrome coronavirus (SARS-CoV), human immunodeficiency virus (HIV) [2] and, notably, SARS-CoV-2 [3]. Vaccines have been a key player in containing the spread and reducing the mortality of bacterial and viral pathogens, taking part in national and global immunization strategies that have led to eradication of smallpox and near eradication of polio [4].

Recombinant viral vectors have become an important platform for vaccination, with growing interest in a variety of possible vectors. Viral vector vaccines have been approved against Ebola [5]—using adenovirus [6], modified Vaccinia Ankara [7,8], and vesicular stomatitis virus [9] as vectors—and against SARS-CoV-2, using adenovirus as a vector (Johnson & Johnson, Gamaleya, Oxford-Astrazeneca and CanSino) [1,10]. There are also examples of approved viral vector vaccines for veterinary use, using vectors such as poxviruses, herpesvirus of turkeys (HVT) [11] and adenovirus [12,13]. This technology fits the concept of platform-based vaccines, in which the viral vector is a backbone that can be modified to express and carry different antigens to quickly adapt the vaccine to target other pathogens, including for emerging outbreaks. By developing a platform-based vaccine and establishing a production process for it, both the product and process can be adapted to other targets with minimal changes. Thus, the time to develop, scale up and, consequently, deliver the vaccine can be greatly reduced, making this a promising approach for pandemic preparedness [14].

In this context, the Newcastle Disease Virus (NDV) is an avian paramyxovirus which is non-pathogenic in humans [2]. NDV is classified into three types of strains: velogenic, mesogenic or lentogenic, based on virulence and pathogenicity in avian species [15]. Lentogenic strains, such as LaSota and B1, are avirulent in both birds and humans, making them the strains of choice for viral vector studies. NDV expresses a fusion protein (F) in its precursor form (F_0_), which must be cleaved by host cell proteases for viral entry [2]. In cell culture, trypsin can be added at the moment of infection to activate the virus [16], as it is done for production of influenza [17]. As an avian virus, there is a lack of pre-existing immunity against NDV among humans, as well as remarkable safety, which has been documented in clinical trials for oncolytic treatments using this virus [2]. Another advantage of using this vector for vaccines includes operation at biosafety level 2, rather than working directly with the target pathogens, which can require costly operation at biosafety level 3 [18]. Additionally, there are well established methods to generate recombinant NDV constructs bearing protective key antigens from other viral pathogens. These aspects make NDV a promising vaccine vector that has already been explored in vaccine candidates against H1N1 influenza, SARS-CoV, HIV, among others [2].

Despite having garnered interest as a viral vector for vaccination and for cancer therapy [15], there are still few studies on the development of production processes for NDV. Typically, this virus is produced in embryonated chicken eggs and collected in the allantoic fluid. Although this is a cost-effective method that can take advantage of existing manufacturing structures for influenza [18], it also presents disadvantages when compared to cell culture technologies and their potential large-scale production under controlled operational conditions in bioreactors. Virus production in cell culture allows for greater control over several parameters, leading to less variation between batches and the capacity to further optimize each aspect. It may also avoid issues with allergens and the dependency on chicken egg supply [19,20]. As such, developing a cell culture-based NDV production process would be highly valuable in the pursuit of establishing a reliable vaccine production platform that allows for quick adaptation to emerging pathogens.

The COVID-19 pandemic highlighted the importance of mass vaccination and the race for fast implementation. Notably, vaccines were developed and approved within months, rather than the usual timespan of years [3], which was partly due to extensive previous research on the technologies used. These vaccines were key to reduce cases and deaths [21], as well as to recover economies [22], in contrast to the 2009 H1N1 pandemic, in which vaccines were only widely available after the main onset of the pandemic [23]. This further showcases the importance of using vaccine platforms to implement vaccination early on in a pandemic, maximizing the positive contribution of vaccines and avoiding the peak of cases and deaths.

In this work, we set out to develop a novel cell-based production process for two NDV constructs: NDV-GFP and the vaccine candidate NDV-FLS, which expresses the full-length SARS-CoV-2 spike protein. We first adapted both constructs to HEK293 and Vero cells, and then evaluated several infection parameters. In shake flasks, viral production kinetics were compared for different multiplicities of infection (MOI) and a Design of Experiment (DoE) was performed to analyze the effects of temperature, trypsin concentration and trypsin addition. The best conditions were then implemented for batch bioreactor productions of both viruses. For the analytics, we established two assays for viral quantification: median tissue culture infectious dose (TCID_50_) for infectious viral titer and digital droplet PCR (ddPCR) for genomic/total viral titer. As such, this is an innovative work in exploring and establishing these essential aspects for robust production and quality assessments of NDV in cell culture.

## 2. Materials and Methods

### 2.1. Cell Lines and Culture Media

The Vero cell line adapted to suspension was provided by the National Research Council of Canada (NRC), and the adaptation was described in a previous work [24]. For routine passaging, cells were centrifuged at 800× *g* for 5 min and resuspended in fresh media with a seeding density of 3–6 × 10^5^ cells/mL. Cell cultures were maintained at 37 °C, 135 rpm and 5% CO_2_ in humified Multitron orbital shakers (Infors HT, Bottmingen, Switzerland). Cells were cultured in MDXK medium (Xell AG, Bielefeld, Germany), supplemented with 4 mM GlutaMAX (Thermo Fisher Scientific, Waltham, MA, USA), at a working volume of 20 mL, 25 mL, 50 mL or 100–200 mL in polycarbonate shake flasks of volume 125 mL, 250 mL, 500 mL or 1 L (TriForest Enterprises, Irvine, CA, USA), respectively.

HEK293 suspension cells are originated from HEK293SF (clone 293SF-3F6) cells, which derive from a GMP-grade master cell bank [25]. The cells were cultured in HEK GM medium (Xell AG, Bielefeld, Germany), supplemented with 4 mM GlutaMAX. Routine passaging and incubation in shakers was the same as for suspension Vero cells.

Adherent Vero cells (ATCC CCL-81.5) were routinely passaged by washing with PBS without calcium and magnesium (WISENT Inc., Saint-Jean-Baptiste, QC, Canada), detaching with TrypLE™ Express Enzyme (Gibco, Gaithersburg, MD, USA) and adding VP Serum-Free Medium (VP-SFM) (Gibco, Gaithersburg, MD, USA) with 4 mM GlutaMAX and 1% Penicillin-Streptomycin solution (WISENT Inc., Saint-Jean-Baptiste, QC, Canada) to collect. Once collected, cells are pelleted at 300× *g* for 5 min and resuspended in VP-SFM to remove TrypLE. Cells are plated onto T-175 flasks or 150 mm plates, at 5–10 × 10^6^ cells and are passaged every 2–3 days.

Adherent HEK293 (HEK293A, ATCC CRL-1573 [26]) cells were routinely passaged in the same way as adherent Vero cells, but using Dulbecco’s Modified Eagle’s Medium (DMEM) (Thermo Fisher Scientific, Waltham, MA, USA) with 10% Fetal Bovine Serum (FBS) (Gibco, Gaithersburg, MD, USA) and 1% Penicillin-Streptomycin solution instead of VP-SFM.

### 2.2. Virus Adaptation

The engineering and rescue of the Newcastle Disease Virus constructs NDV-GFP and NDV-FLS were described in another publication [27]. Briefly, the gene of interest (encoding green fluorescent protein or human codon-optimized full-length spike from SARS-CoV-2, respectively) was inserted between the P and M genes of the NDV (LaSota strain) genome. These viruses were initially produced in allantoic fluid and passaged for adaptation to Vero and HEK293 cells. Passages consisted of infecting cells, harvesting the virus produced and using it to reinfect cells for the next passage.

In Vero cells, for passages 1 and 2, adherent Vero cell cultures in T-25 flasks with VP-SFM media and 4 mM GlutaMAX were infected at a confluency of 80–90% and an MOI of 0.5 with TPCK-treated trypsin (MilliporeSigma, Oakville, ON, Canada) to a final concentration of 1 μg/mL. The supernatant was collected at 24 hpi. From passage 3 onwards, suspension Vero cells were seeded at 1 × 10^6^ cells/mL in 25 mL MDXK media with 4 mM GlutaMAX in 250 mL shake flasks. The cells were immediately infected at an MOI of 0.01 with 1 μg/mL trypsin. At 36 hpi, the culture was centrifuged at 800× *g* for 5 min to collect the supernatant, which was stored at −80 °C.

In HEK293 cells, for all passages, suspension cells were seeded at 1 × 10^6^ cells/mL in 25 mL Xell HEK GM media with 4 mM GlutaMAX in 250 mL shake flasks. The cells were immediately infected at an MOI of 0.01 with 1 μg/mL trypsin. At 36 hpi, the culture was centrifuged at 800× *g* for 5 min to collect the supernatant, which was stored at −80 °C.

### 2.3. Median Tissue Culture Infectious Dose (TCID_50_)

For routine quantification, adherent Vero cells were seeded on 96-well plates with 15,000 cells in 100 μL of media (VP-SFM) per well. For media and cell line comparison during TCID_50_ development, adherent HEK293 cells were used with DMEM. When using DMEM, BSA 2.5 μg/mL was added instead of FBS, to avoid trypsin activity inhibition. The following day, the media was aspirated and replaced by 100 μL of media containing 1 μg/mL trypsin and a serial dilution of the virus (1:5 or 1:10). After 4 and 7 days of incubation at 37 °C with 5% CO_2_, wells were analyzed on a standard light microscope for cytopathic effect (CPE), consisting of rounded cells, a disrupted monolayer and/or clumps. The number of CPE-positive wells in each column was used to quantify the experiment by the Spearman and Kärber algorithm [28,29,30].

The assay with 1:5 dilutions (Coefficient of Variation: 34.57%) was chosen for all the TCID_50_ development and for samples which were below the range of detection of the 1:10 dilutions (<3.16 × 10^2^ TCID_50_/mL). The assay with 1:10 dilutions (Coefficient of Variation: 34.69%) was chosen for all samples from shake flask experiments and bioreactors.

For comparison of CPE readings and Alamar blue readings, CPE was read first on the microscope before addition of the dye. The cell viability reagent Alamar blue (Invitrogen, Waltham, MA, USA) was diluted 1:10 in PBS without calcium and magnesium, and 100 µL of the dilution was added to each well, as described previously [31]. Plates were incubated at 37 °C with 5% CO_2_ and the absorbance was analyzed after 4 h. The absorbances at 570 nm and 600 nm were measured, and the absorbance at 600 nm was subtracted from the absorbance at 570 nm (ABS_570nm_ − ABS_600nm_) to obtain the normalized value. Cut-off values were determined in a way that none of the wells in the (non-infected) negative control would be considered infected.

For comparison with fluorescence readings, a triplicate of an NDV-GFP sample was used for TCID_50_ and plates were read both by CPE, using a standard light microscope, and by fluorescence, using a plate reader with the excitation at 485/20 nm and emission at 528/20 nm.

After classifying the wells as positive through the cell viability (Alamar blue) or the fluorescence, the viral titer was determined by the Spearman and Kärber algorithm [28,29,30], in the same way as when reading CPE.

For fluorescent microscope imaging, the TCID_50_ plates infected with NDV-GFP samples were observed on day 7 on Olympus IX-83 microscope using a 10× objective lens. Images were processed on ImageJ to merge bright-field images with green fluorescence channel images.

One-way ANOVA with the Tukey method was performed to determine statistical significance when comparing titration between different cell lines and different reading methods.

### 2.4. Polymerase Chain Reaction (PCR)

The Q5 High Fidelity Polymerase (New England Biolabs, Ipswich, MA, USA) was used with primers targeting the L gene (polymerase) of NDV: NDV-L F [5′-ATATGTTCTGACTCCTGCCC-3′] and NDV-L R [5′-TCTAGTCGCTTGATCTCTGC-3′]. PCR was performed according to the manufacturer’s instructions, with the following thermocycler program: initial denaturation (1 min at 98 °C), followed by 30 cycles of the steps: 10 s at 98 °C, 30 s at the annealing temperature, 30 s at 72 °C. Next, the final elongation step happens for 2 min at 72 °C. The same NDV-GFP and NDV-FLS cDNA samples were used for PCR with different annealing temperatures: 56 °C, 57.6 °C, 59.2 °C and 60 °C. The amplified bands were visualized in a 2.5% agarose gel with SYBR Safe DNA gel stain (Thermofisher, Waltham, MA, USA).

### 2.5. Digital Droplet Polymerase Chain Reaction (ddPCR)

For routine quantification, RNA extraction was done for 20 µL of supernatant samples diluted with 180 µL PBS (without calcium and magnesium) using the High Pure Viral Nucleic Acid kit (Roche, Basel, Switzerland). During assay development, different dilutions of the sample were also tested: 1× (no dilution—200 µL sample), 4× (50 µL sample with 150 µL PBS) and 10× (20 µL sample with 180 µL PBS). Next, 2 µL of the extracted RNA was used with the iScript Select cDNA synthesis kit (Bio-Rad Laboratories, Hercules, CA, USA) to generate cDNA using random RT-PCR primers. Then, the cDNA was diluted (between 1:10 to 1:10,000) to target the linear range of ddPCR. 5 µL of the template dilution was used with the QX200 ddPCR kit (Bio-Rad Laboratories, Hercules, CA, USA), using the EvaGreen master mix and the same primers listed for PCR. The manufacturer’s instructions were followed to prepare the reaction and generate droplets. As for the thermocycler program: after initial denaturation (5 min at 95 °C), 34 cycles of the following steps were repeated: 30 s at 95 °C, 1 min at 59 °C, 30 s at 72 °C. Then, the final elongation step happened for 5 min at 72 °C.

Droplets are analyzed individually in the droplet reader and the copies/µL of each sample is given. This output is corrected for the dilution and volumes used to determine the viral genomes/mL of the original sample with the following calculation:

Viral genomes/mL = I × J × K × (L/M) × (O/N)/P × Q × 1000,

in which: I = Copies/µL (ddPCR output); J = volume of the ddPCR reaction; K = dilution of the cDNA template; L = volume of RT-PCR reaction; M = volume of the cDNA dilution added in the ddPCR reaction; N = volume of RNA added in the RT-PCR reaction; O = elution volume for RNA extraction; P = initial sample volume used for the RNA extraction; Q = dilution of the sample in RNA extraction.

### 2.6. Design of Experiment (DoE) for Infection Parameters

A two-level full factorial design was done with triplicates of each condition to screen 3 parameters at infection: trypsin concentration (from 1 to 5 µg/mL), trypsin addition (no repeated addition or addition at 24 h) and temperature (from 34 to 37 °C). To start the experiment, cultures of suspension Vero cells were centrifuged at 800× *g* for 5 min and seeded at 1 × 10^6^ cells/mL in 30 mL MDXK media with 4 mM GlutaMAX in 250 mL shake flasks. The flasks were immediately infected with NDV-FLS at an MOI of 0.01 using the chosen DoE parameters. For trypsin addition at 24 h, trypsin was added to a final concentration of 1 or 5 µg/mL, according to the initial trypsin concentration assigned to each flask. Viral samples were taken at 30 hpi by centrifuging at 800× *g* for 5 min and aliquoting the supernatant (storage at −80 °C). Samples were quantified by TCID_50_ and analyzed with the Design Expert 13 software (Stat-Ease Inc., Minneapolis, MN, USA) using base 10 log-transformed values. Statistical significance was determined through ANOVA, followed by several residual analyses and diagnostics to confirm the quality of the model.

### 2.7. Multiplicity of Infection (MOI) Optimization

4 different multiplicities of infection (MOI) were evaluated: 0.1, 0.01, 0.001 and 0.0001 IVP/cell. Cultures of suspension Vero cells were centrifuged at 800× *g* for 5 min and seeded at 1 × 10^6^ cells/mL in 25 mL MDXK media with 4 mM GlutaMAX in 250 mL shake flasks. Immediately, cells were infected with NDV-FLS at the corresponding MOIs with 1 µg/mL trypsin, in triplicates. Shake flasks were incubated at 37 °C, 135 rpm and 5% CO_2_ and samples were taken every 12 h after infection. For sampling, 0.8 mL culture of each flask was taken and spun down at 800× *g* for 5 min to aliquot the supernatant (storage at −80 °C). Viral samples were quantified by TCID_50_ and triplicates were averaged to plot the viral production kinetics of each MOI. The peak values of viral production for each MOI were compared in a one-way ANOVA with the Tukey method to investigate statistical significance.

### 2.8. Bioreactors

For 1 L bioreactors, a culture of 650 to 850 mL was seeded at 2.5 × 10^5^ cells/mL in MDXK with 4 mM glutamine. The bioreactors (Applikon Biotechnology, Delft, The Netherlands) were assembled with a marine impeller for stirring and sensors for dissolved oxygen (DO) concentration, temperature and pH. The following parameters were controlled in the system: pH at 7.2, temperature at 37 °C, DO at 40–50% and stirring at 100 rpm. DO was maintained by a constant airflow in the headspace of 10 mL/min, along with pure oxygen sparging when necessary. The pH was regulated by addition of CO_2_ in the headspace or injection of NaHCO_3_ (90 g/L) (Sigma, USA).

Samples were taken every 24 h to monitor cell growth using the Vi-CELL XR Cell Viability Analyzer (Beckman Coulter Life Sciences, Brea, CA, USA). Glutamine was injected at 1 mM daily. For virus production, cells were infected at an MOI of 0.01 IVP/cell with 1 µg/mL trypsin on day 4 or 5 of culture, when cell density was approximately 8 × 10^5^ cells/mL. After infection, samples were taken every 12 h and the supernatant was obtained by centrifuging at 800× *g* for 5 min (storage at −80 °C).

## 3. Results

### 3.1. Development and Optimization of Analytical Assays

#### 3.1.1. Cell Line Assessment for TCID_50_ Set-Up

**Figure 1:**
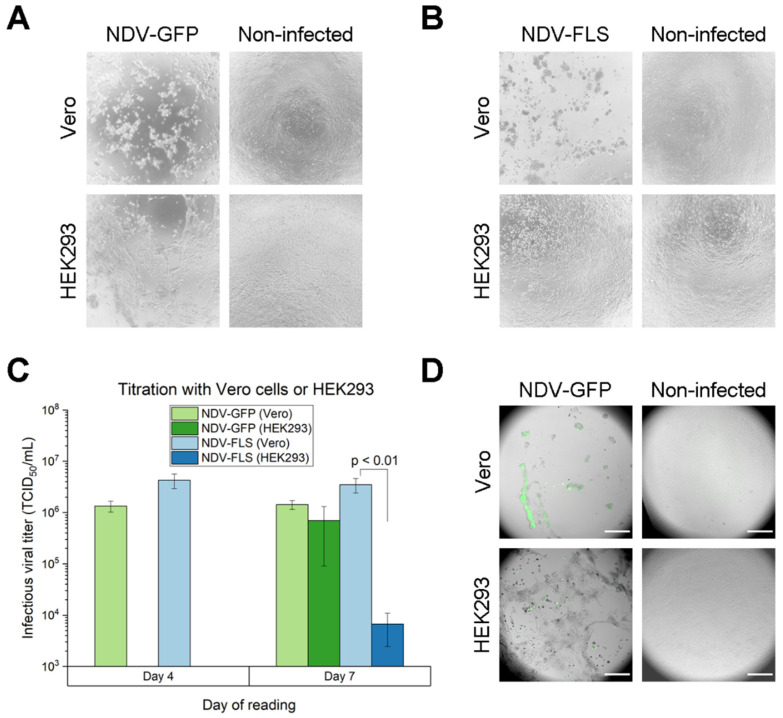
Comparison between Vero cells in VP-SFM or HEK293 in DMEM for quantification by TCID_50_ of NDV-GFP and NDV-FLS viruses. (**A**,**B**) Cytopathic effect seen in Vero and HEK293 adherent cells after 7 days of incubation with the two different viruses: NDV-GFP (**A**) and NDV-FLS (**B**). The comparisons show an infected well in TCID_50_ and a negative control well, with no virus. (**C**) Infectious titers obtained by quantifying the same NDV-GFP and NDV-FLS viral samples using Vero or HEK293 cells for TCID_50_. Error bars correspond to the average of triplicate plates ± standard deviation. Statistically significant differences are labeled with the corresponding *p*-value. (**D**) Fluorescence detection in TCID_50_ wells infected with NDV-GFP in Vero or HEK293 cells, compared with the respective non-infected negative controls. The images show a merge of the green fluorescence channel (GFP) with the bright-field image (cells). Scale bar corresponds to 250 µm.

Adherent Vero and HEK293 cells were compared in order to choose the most adequate cell line for the development of a TCID_50_ assay, to quantify infectious viral particles in culture supernatants. When using NDV-GFP and NDV-FLS viral samples to infect both cell lines in TCID_50_ assays, evident differences were observed upon visual inspection of cytopathic effect, as well as in the measured titers and fluorescence detection (Figure 1). These TCID_50_ plates were incubated for 7 days after infection, and analyzed on days 4 and 7.

When comparing the cytopathic effect generated on each cell line by the same virus inoculum, NDV-GFP induced a strong effect in Vero and HEK293, forming large clumps of non-viable cells and completely disrupting the cell monolayer after 7 days of incubation (Figure 1A). However, with NDV-FLS, the cytopathic effect was more evident in Vero cells than in HEK293 (Figure 1B). Like NDV-GFP, it caused the formation of aggregates and disrupted the Vero cell monolayer, but in HEK293 the cytopathic effect was limited to individual rounded cells, with very rare clumping. Even in Vero cells, NDV-FLS showed a slightly smaller cytopathic effect when compared to NDV-GFP (Figure S1), forming smaller aggregates that were still clearly distinguishable from the non-infected cells. When comparing different media for adherent Vero, cytopathic effect for both viruses was more noticeable when using VP-SFM (Figure S1). DF-1 cells were also tested in the TCID_50_ assay, but cytopathic effect was not discernible compared to non-infected cells, even after 7 days of incubation.

For both viruses, 4 days of incubation in HEK293 was not enough to allow for quantification, as the cytopathic effect was still difficult to distinguish from non-infected cells. This was only possible on day 7 of incubation (Figure 1C). With Vero cells, on the other hand, cytopathic effect was already distinguishable on day 4, allowing for an earlier quantification. When comparing the quantification for the same viral sample in the different cell lines on day 7, NDV-GFP had no significant differences, but the titer for NDV-FLS obtained with Vero cells was significantly higher (*p* < 0.01) than with HEK293. This was in line with the more subtle cytopathic effect observed with NDV-FLS in HEK293, which resulted in a more difficult reading and apparent lower titers. Since both constructs came from egg-derived aliquots with similar yielding passages, the titers observed when quantifying with Vero cells were more adequate, with both constructs resulting in similar titers.

Lastly, the TCID_50_ plates infected with NDV-GFP were imaged under an inverted confocal fluorescence microscope. In Vero cells, the aggregates seen in the cytopathic effect were paired with strong fluorescence (Figure 1D). In HEK293, however, there was less fluorescence, even when abundant cytopathic effect was present. Although NDV-GFP showed signs of infection in both cell lines, GFP production was higher in Vero cells.

When analyzing all three aspects (cytopathic effect, titers and fluorescence), Vero cells seemed to be more suitable for NDV titration than HEK293 cells, with distinguishable cytopathic effect, higher titer and fluorescence, aside from allowing quantification within a shorter period of time. Thus, adherent Vero cells were chosen as the most appropriate cell line for the TCID_50_ assay and were used in all subsequent quantifications.

#### 3.1.2. Quantification of NDV Infectious Particles through Fluorescence Measurements and Viability-Based Assays

**Figure 2:**
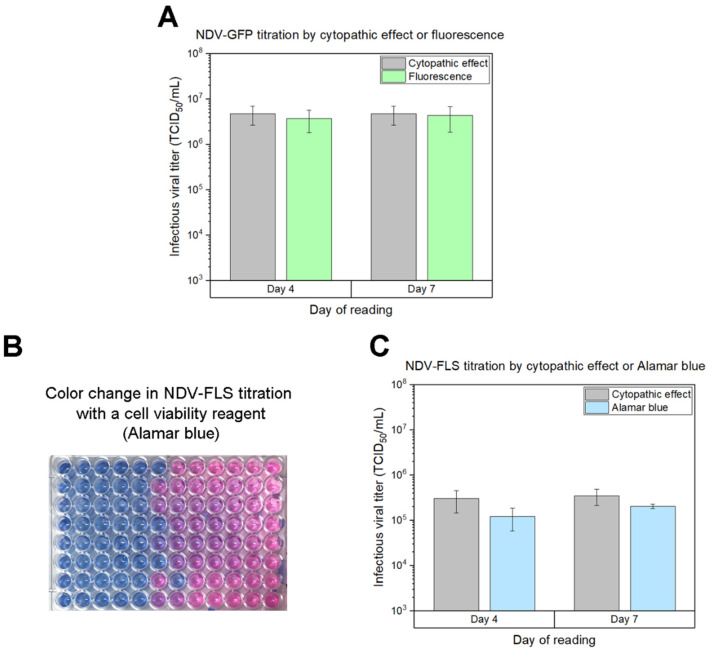
Different titration assays for NDV infectious particle determination. (**A**) Titration of the same sample of NDV-GFP in triplicate quantified by CPE and by fluorescence. Error bars correspond to the average of triplicate plates ± standard deviation. (**B**) TCID_50_ plate (on day 7) after 4 h of incubation with a cell viability reagent (Alamar blue). Blue wells corresponded to infected/dead cells (low viability) while pink wells corresponded to non-infected/healthy cells (high viability). (**C**) Titration of the same sample of NDV-FLS in triplicates quantified by CPE and by the cell viability reagent Alamar blue. Error bars correspond to the average of triplicate plates ± standard deviation.

The next step in TCID_50_ development was to use a plate reader to test alternative methods of reading, which do not require subjectively analyzing cytopathic effect on a microscope. For NDV-GFP, the green fluorescence was read on a plate reader to determine the infected wells and calculate the infectious titer (Figure 2A). When quantifying the same sample by cytopathic effect or by fluorescence, there was no statistically significant difference between the two methods, both on day 4 and day 7 (*p* = 0.5653 and *p* = 0.8301, respectively). This showed that fluorescence can also be used for quantification and that the virus infected the cells, simultaneously expressing detectable GFP. Most wells with cytopathic effect also showed fluorescence on days 4 and 7 (95.48% and 98.92%, respectively).

Since fluorescence can only be used to quantify NDV constructs bearing the GFP coding sequence, a reading method based on cell viability was also evaluated. For TCID_50_ calculations, the plates were incubated with a cell viability reagent (Alamar blue), resulting in infected wells that remained blue while the non-infected ones, containing healthy cells, became red/pink (Figure 2B). The infectious titer of the same NDV-FLS sample was quantified by cytopathic effect observation on the microscope and by cell viability staining, resulting in similar titers and no statistically significant differences between both methods on day 4 and day 7 (*p* = 0.1395 and *p* = 0.1478, respectively) (Figure 2C).

#### 3.1.3. ddPCR-Based Quantification of NDV

**Figure 3:**
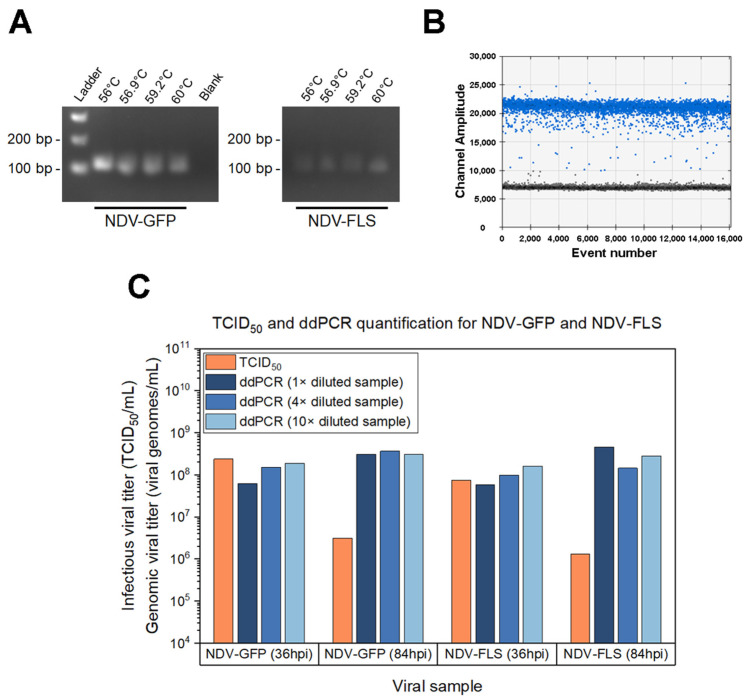
Development of a digital droplet PCR (ddPCR) assay for quantification of NDV. (**A**) Agarose DNA gel to verify PCR reactions at different annealing temperatures with primers designed for ddPCR, targeting the NDV-L (polymerase) gene on an NDV-GFP and an NDV-FLS sample. The expected band is 117 bp. (**B**) Plot showing positive (blue) and negative (dark grey) events in ddPCR. (**C**) Comparison between each sample’s infectious titer (TCID_50_/mL) with the genomic titer (viral genomes/mL) quantified by ddPCR. For ddPCR, different dilutions of the viral sample in RNA extraction were used: a non-diluted sample (1×), a 4 times diluted sample (4×) and a 10 times diluted sample (10×). Sample dilutions were taken into account in the calculation of final titers. Samples of NDV-FLS and NDV-GFP at a peak production time point (36 h post infection) and late time point (84 h post infection) were used.

A quantification assay based on digital droplet PCR (ddPCR) was developed to measure total viral particles. First, different annealing temperatures were tested by PCR to confirm specificity, using NDV-GFP and NDV-FLS samples (Figure 3A). For all temperatures tested with both viruses, the expected amplification product was observed, without presence of non-specific bands.

Next, the chosen primers were used for ddPCR, with the selected annealing temperature of 59 °C. Individually partitioned events were clearly defined as positive or negative (Figure 3B), indicating proper functioning of the assay. When performing ddPCR on viral samples from peak production time points (36 hpi), the genomic titer was similar or higher than the infectious titer quantified by TCID_50_ (Figure 3C). For later time points (84 hpi), the genomic titer was notably higher than the infectious titer, as the infectious titer decreased, and the genomic titer remained constant. Out of the three sample dilutions for the viral supernatant tested in the RNA extraction step, the 10× dilution was selected for the genomic quantification of NDV in subsequent experiments.

### 3.2. Evaluation of NDV Infection and Production Parameters

**Figure 4:**
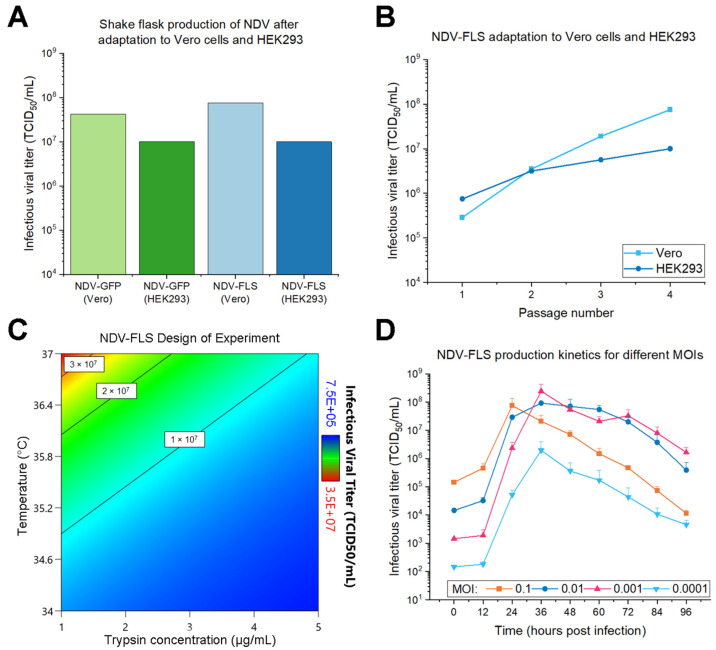
Optimization of infection parameters in small scale shake flask productions. (**A**) Infectious viral titers achieved in the fourth round of infection (passage 4) of each NDV construct in the two cell lines evaluated. (**B**) Serial passaging of NDV-FLS in Vero and HEK293 cells for viral adaptation. The first two passages in Vero were conducted in adherent cells with MOI = 0.5. Passages 3 and 4 were conducted in suspension cells with MOI = 0.01. For HEK293, suspension cells infected at MOI = 0.01 were used in all the passages. (**C**) Design of experiment (DoE) modeling for production of NDV-FLS, showing the highest viral titer produced at 37 °C with 1 µg/mL trypsin added to the culture media. (**D**) Viral production kinetics for NDV-FLS using the MOIs 0.1 to 0.0001, with the highest titer achieved of around 1.00 × 10^8^ TCID_50_/mL. Time is shown as hours post infection (hpi). Error bars correspond to the average titer calculated from shake flask triplicates + standard deviation.

The two viral constructs, which were initially produced in eggs and contained in allantoic fluid, were serially passaged in Vero and HEK293 cell lines for adaptation (Figure 4A,B).

For both NDV-GFP and NDV-FLS, higher infectious titers were achieved in Vero than in HEK293 cells after adaptation. Viral production in Vero cells at passage 4 was 4.22 × 10^7^ TCID_50_/mL for NDV-GFP and 7.50 × 10^7^ TCID_50_/mL for NDV-FLS, while production in HEK293 reached a maximum of 1.00 × 10^7^ TCID_50_/mL for both viruses (Figure 4A). As shown for NDV-FLS (Figure 4B), both cell lines started with productions lower than 3 × 10^6^ TCID_50_/mL at passage 1 and showed increased viral titers as passages progressed. This increase throughout adaptation was higher in Vero cells (over 250-fold) than in HEK293 (less than 20-fold). After passage 4, subsequent passaging for NDV-FLS or NDV-GFP did not increase the titer levels. Thus, suspension Vero cells were selected for NDV production and further optimizations.

Next, a two-level full factorial design of experiment was done to determine parameters for infection with NDV-FLS (Figure 4C). Temperature (*p* < 0.0001) and trypsin concentration at infection (*p* = 0.0004) impacted infectious titers significantly, with the best condition being 1 µg/mL of trypsin and incubation at 37 °C. The third parameter, however, which was trypsin addition at 24 h post infection vs. no repeated addition, showed no statistically significant difference (*p* = 0.3271). As such, the best conditions were used for the next experiments, with no repeated trypsin addition.

Additionally, different MOIs were tested for NDV-FLS, ranging from 0.1 to 0.0001 (Figure 4D). The lowest MOI (0.0001) had the lowest peak of viral production (1.96 × 10^6^ TCID_50_/mL), while the other 3 MOIs (0.1–0.001) all reached similar peaks around 1.00 × 10^8^ TCID_50_/mL, with no significant differences (*p* = 0.178). As expected, the highest MOI showed the earliest peak, at 24 hpi, while the next two MOIs (0.01 and 0.001) peaked at 36 hpi. Despite having an earlier peak, the infectious titer with MOI 0.1 dropped considerably as time progressed to 96 hpi, declining to similar titers as those reached at the lowest MOI. Although the MOIs 0.01 and 0.001 also showed a loss in infectious titer after the peak, the losses were the smallest when compared to other MOIs. Therefore, the MOI 0.01 was chosen for the following viral productions, with a high peak of production and adequate stability. Upon applying the selected conditions to the NDV-GFP construct, the titer of 1.07 × 10^8^ TCID_50_/mL was obtained in shake flasks.

### 3.3. Production in Bioreactors

**Figure 5:**
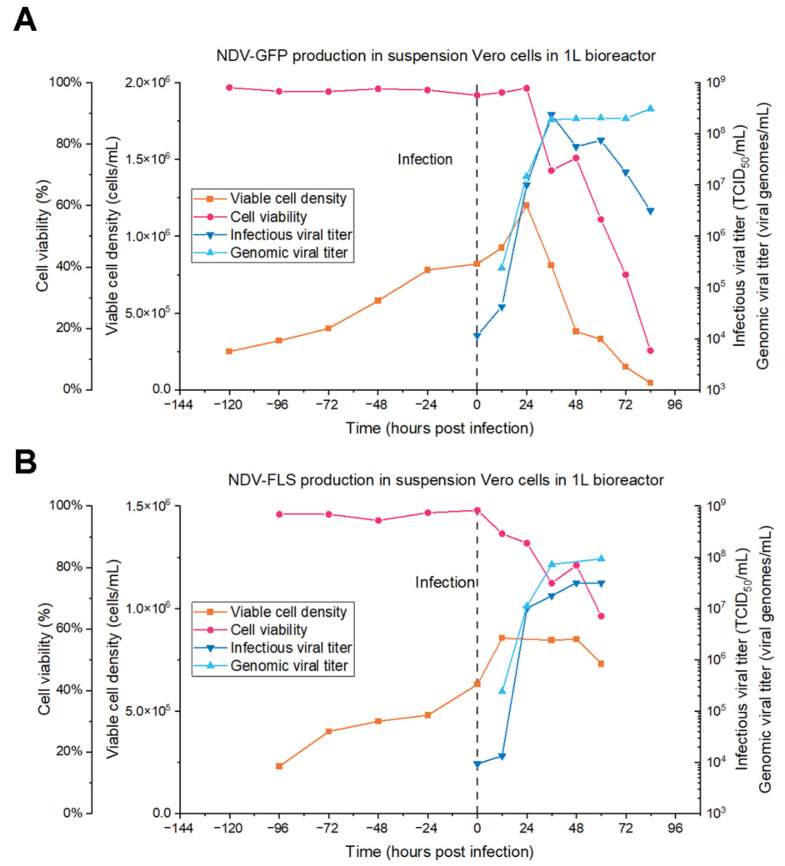
Batch bioreactor production of NDV-GFP (**A**) and NDV-FLS (**B**) at the 1 L scale. Offline measurements were taken by regular sampling. Infectious viral titers were quantified by TCID_50_ and total/genomic viral titers were quantified by ddPCR. The time of infection is indicated by a black dashed line in the figure.

After parameter optimization in shake flasks, the next aim was to produce the viruses in suspension Vero cells using stirred tank bioreactors. A 1 L batch bioreactor was performed for production of NDV-GFP (Figure 5A) and NDV-FLS (Figure 5B). Infectious titers quantified for both viruses showed the ability of the system to reach peaks in the orders of 10^8^ and 10^7^ TCID_50_/mL, respectively.

For NDV-GFP (Figure 5A), the infectious titers peaked at 36 hpi, reaching 2.37 × 10^8^ TCID_50_/mL, after which values decreased over time, dropping to 3.16 × 10^6^ TCID_50_/mL at 84 hpi. The total viral titer, on the other hand, remained constant after the peak production, at around 2.00 × 10^8^ VGs/mL. The virus also affected cell viability, as seen with the considerable drop to below 80% observed at 36 hpi, that reached below 20% by the end of the bioreactor run at 84 hpi.

For NDV-FLS (Figure 5B), the peak production was 3.16 × 10^7^ TCID_50_/mL at 48 hpi, which remained constant until 60 hpi. The genomic titer was higher than the infectious titer, plateauing at around 1 × 10^8^ VGs/mL from the peak production onwards. A decrease in cell viability was observed post infection, dropping to lower than 65% at 60 hpi.

**Figure 6:**
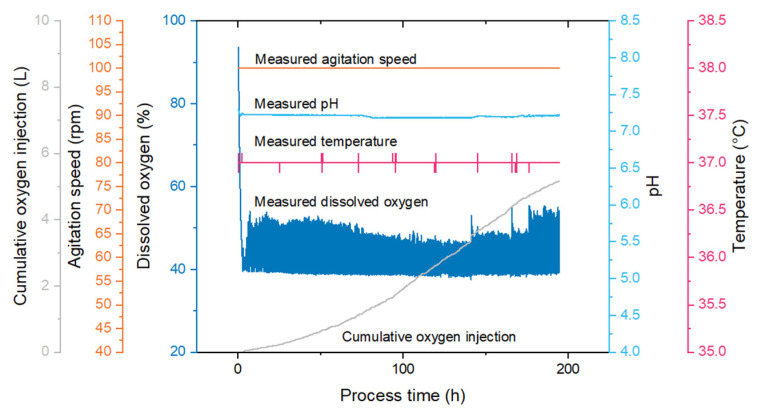
Online bioreactor measurements recorded throughout a batch bioreactor production of NDV-FLS at the 1 L scale.

The online measurements for bioreactor productions of NDV showed that pH, temperature and DO were maintained constant during the cell growth and virus production phases (Figure 6) through effective control strategies, including the addition of oxygen.

## 4. Discussion

NDV is a promising viral vector for vaccine development that has been studied for its potential application against several human diseases, and it is still commonly produced in embryonated chicken eggs [2]. In this study, we set out to provide an alternative for NDV production by developing the foundation for a cell culture-based production process. Bioprocesses for vaccine manufacturing are composed of an upstream phase, a downstream phase, and the analytics used throughout the entire process to quantify the production and optimizations. Here, we developed analytical assays and evaluated upstream process parameters by testing cell lines for production, adapting the virus to suspension cell cultures and comparing several infection conditions. After this evaluation, we applied the selected parameters to produce NDV in 1 L scale bioreactors.

MDCK and Vero cells are well established systems for viral vaccine production, but a range of other continuous cell lines (CCLs) have also been studied for this purpose, including HEK293. While HEK293 shows promise as a cell line that has been adapted for suspension and grows to high densities in serum-free media [32], manufacturers tend to prefer processes using established cells for faster licensing [33]. Vero cells have a long history of proven safety, being the first CCL approved for viral vaccine production for human use. From a process perspective, these cells are commonly used with adherent cell culture technologies, such as microcarriers or fixed bed bioreactors, which are labor intensive and limited by surface area, resulting in a difficult scale up [20]. However, recent advances in adapting Vero cells to suspension have been successful [24], as these suspension Vero cells have been shown to work for virus production using stirred tank bioreactors in batch [34] and perfusion [24] modes. These cells have also been adapted to grow in the serum-free commercially available MDXK medium, after screening with several other media [34]. Recently, this cell line’s genome has been sequenced through de novo assembly and annotated, facilitating future genome editing approaches [35]. Furthermore, Vero cells are interferon-deficient [35], making them susceptible to a wide range of viruses that have achieved high productivity when produced in Vero cells [20].

In this study, suspension Vero cells showed the additional ability of yielding higher viral titers for both NDV-GFP and NDV-FLS constructs, which was in line with the more evident CPE and intensity of fluorescence observed in adherent Vero cells when compared to HEK293. Serial passaging of NDV in Vero cells led to an increase in titer after four passages, similar to what has been shown for other strains of NDV [36], in which the number of passages required for such an increase varied for each strain. This increase is expected, as the viruses were originally collected in allantoic fluid, and viral adaptation to cell culture may select for viruses with more efficient replication in the new host cell. Further characterization of the viruses adapted to these cell lines could be important to evaluate if there were changes to safety, efficacy and abundance of recombinant protein on the viral surface when compared to the virus produced in eggs.

After defining suspension Vero as the cell line of choice for NDV production, a DoE revealed that the highest NDV-FLS titers were obtained when infecting at 37 °C with 1 µg/mL trypsin, and that repeated trypsin addition had no significant effect. VSV titers are influenced by the temperature in the production phase, and each construct has an optimal temperature [34]. As the LaSota strain of NDV is not thermostable [37], similarly to VSV, a lower temperature could have resulted in higher viral titers. However, a production temperature of 37 °C led to significantly higher titers than 34 °C, ruling out the use of low temperatures for these NDV constructs. This may be in line with the 37 °C incubation step that is typically implemented when producing NDV in embryonated eggs [18,38]. As for trypsin, the concentrations tested were 1 and 5 µg/mL, which are values reported in the literature for NDV experiments [37,39]. In our study, the highest NDV titers were achieved with the lowest trypsin concentration, which is similar to what has been observed for influenza virus [17]. Vero cells are known to produce trypsin inhibitors [40], and multiple additions of trypsin have been described as having a positive effect [41] or no effect [40] on the multi-cycle production of influenza in this cell line. For NDV, we found that repeated trypsin addition had no apparent effect on the viral titer produced, which prompted us to add trypsin only at the moment of infection.

A range of MOIs (0.1–0.0001) that encompasses the MOIs used for NDV in previous works [37,39,42] was also evaluated. With the exception of the lowest one tested, all MOIs reached a similar peak of approximately 1 × 10^8^ TCID_50_/mL. The viral production peak was 24 hpi for the highest MOI (0.1), and shifted to a later time point (36 hpi) with lower MOIs. However, this higher MOI showed a greater and earlier loss of infectivity than the next two MOIs assayed (0.01 and 0.001). For the 0.01 MOI, the titer remained relatively constant until 60 hpi, and was still higher than the 0.1 MOI by the end of the experiment at 96 hpi. Such stability is important for a robust process, as it is more likely to result in an adequate yield even if production kinetics shift due to variations in the process. The 0.01 MOI was chosen for the process, since an MOI 10 times lower still yielded similar results, and thus possible volume errors when adding the virus at 0.01 MOI would still lead to a reliable production. Overall, from the first passage in Vero cells to the last shake flask optimization experiment, the produced NDV-FLS titers increased by almost 320-fold, from 2.87 × 10^5^ TCID_50_/mL to 9.17 × 10^7^ TCID_50_/mL, indicating that the selection of culture and infection parameters was adequate.

Aside from the cell lines and infection parameters used, analytics are also an essential part of the production process that should not be overlooked. The virus being produced must be characterized and quantified throughout several steps of manufacturing to generate crucial data for process development and for regulatory approval [43]. As a replicative viral vector [2], NDV can be quantified regaring the replication-competent particles—also known as functional or infectious titer—and regarding the total particles, which may or may not be functional. The ratio between these two titers is indicative of quality and can be used to assess different time points or conditions of the process [44]. As such, when developing a process, it is important to establish reliable and scalable analytical methods to increase feasibility of implementing this process in large scale, consequently improving the chances of quickly achieving mass vaccination for a new pathogen of concern.

In this study, not only have we developed assays for each type of quantification, but we have also established methods of reading the TCID_50_ assay amenable to automation. NDV-GFP was quantified by reading fluorescence on a plate reader, while other constructs, such as NDV-FLS, can also be quantified on a plate reader when paired with a reagent that detects viability. Alamar blue is a blue dye based on resazurin, which changes to a red color when reduced to resorufin in metabolically active cells, indicating cell health [45]. Both fluorescence and viability were shown to be comparable to CPE quantification, resulting in valid methods of reading TCID_50_. As these methods rely on plate readers, and not visual inspection, they are non-subjective and can be automated for use in industry or for standardization across collaborating institutions and facilities. Therefore, the availability of these tools makes the assay more feasible for high throughput processes and industrial application. Antibody-based assays, such as an immunofluorescence assay (IFA) [18,46], could also be of interest, as they can be targeted to quantify only viruses that contain the protein required for immunization, which could be important in vaccine manufacturing. This specificity, however, means having to adapt the assay with a different antibody for each new construct, which could slow down the development of new vaccines using the platform. Therefore, TCID_50_ and ddPCR assays were chosen, as they can be used for any NDV construct.

After establishing the infection parameters at small scale and the analytical assays, we set out to produce NDV in batch mode in 1 L stirred tank bioreactors. For NDV-GFP, the peak titer produced was 2.37 ± 0.82 × 10^8^ TCID_50_/mL at 36 hpi, which is similar to the highest titer observed in shake flasks (1.07 ± 0.37 × 10^8^ TCID_50_/mL). As for NDV-FLS, the peak production was 3.16 ± 1.09 × 10^7^ TCID_50_/mL at 48 hpi, which is similar to the value at 36 hpi (1.78 ± 0.62 × 10^7^ TCID_50_/mL) when considering the analytical error. This is lower than the highest values achieved with this MOI in shake flasks (9.17 ± 1.44 × 10^7^ TCID_50_/mL), which can occur when scaling up to bioreactors because of differences in stirring and many other factors. Both productions are comparable to the titers produced in embryonated eggs, which is in the order of 10^8^ FFU/mL [18] and 10^7^ PFU/mL [47], indicating that the bioreactor-based process developed in this study is a valuable substitute for existing egg-based productions.

Furthermore, process intensification could increase the quantity of infective particles harvested, using technologies such as fed-batch or perfusion [48]. The lower infectious titers observed at later time points in bioreactors and shake flasks suggest that NDV could be a good candidate for production in perfusion mode, as the viruses could be continuously harvested before suffering a loss in infectivity due to temperature and shear stress in the bioreactor.

Therefore, we have successfully developed the upstream process and analytical methods for suspension Vero cell-based production of NDV, using the constructs NDV-GFP and NDV-FLS as models. Future steps include establishing a scalable purification protocol and testing different bioreactor production modes, such as fed-batch and perfusion, to move towards a complete process based on continuous manufacturing.

## Data Availability

The data presented will be made available through the corresponding author upon request.

## Supplementary Materials

The following are available online at https://www.mdpi.com/article/10.3390/vaccines9111335/s1, Figure S1: Cytopathic effect seen in TCID_50_ when using different media with Vero cells.
