# Supplementary material for: Process Development for Newcastle Disease Virus-Vectored Vaccines in Serum-Free Vero Cell Suspension Cultures
Source: Vaccines (Basel). 2021 Nov 16;9(11):1335. doi: 10.3390/vaccines9111335 (PMC8623276; doi:10.3390/vaccines9111335)
Supplement: Supplementary file 1 [file vaccines-09-01335-s001.zip › vaccines-1448247-supplementary.pdf]

Supplementary data

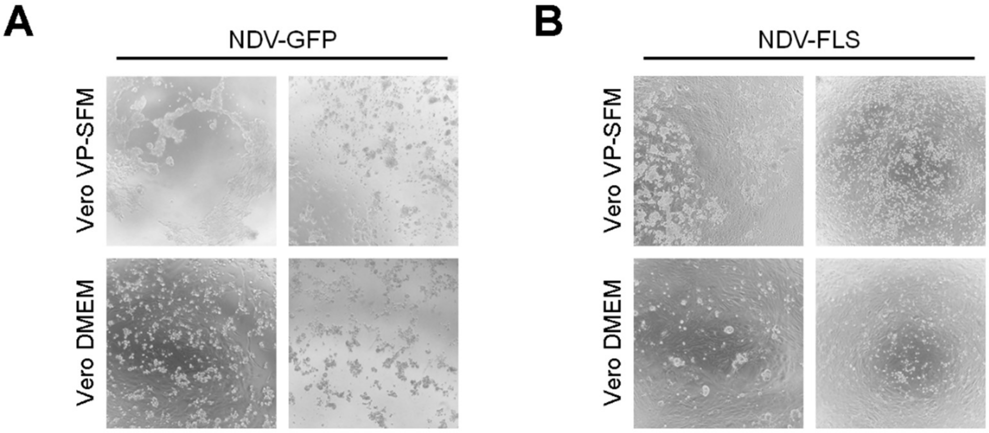

Figure S1. Cytopathic effect seen in $TCID_{50}$ assays when using different media for Vero cells. **A)** Cytopathic effect seen in $TCID_{50}$ assays using Vero cells infected with NDV-GFP, in either VP-SFM or DMEM media. **B)** Cytopathic effect seen in $TCID_{50}$ infected with NDV-FLS, using Vero with either VP-SFM or DMEM.
